# Preparation of Triple-Negative Breast Cancer Vaccine through Electrofusion with Day-3 Dendritic Cells

**DOI:** 10.1371/journal.pone.0102197

**Published:** 2014-07-18

**Authors:** Peng Zhang, Shuhong Yi, Xi Li, Ruilei Liu, Hua Jiang, Zenan Huang, Yu Liu, Juekun Wu, Yong Huang

**Affiliations:** 1 Department of Thyroid and Breast Surgery, The Third Affiliated Hospital of Sun Yat-sen University, Guangzhou, Guangdong, China; 2 Department of Hepatic Surgery, The Third Affiliated Hospital of Sun Yat-sen University, Guangzhou, Guangdong, China; University of Missouri-Kansas City, United States of America

## Abstract

Dendritic cells (DCs) are professional antigen-presenting cells (APCs) in human immune system. DC-based tumor vaccine has met with some success in specific malignancies, inclusive of breast cancer. In this study, we electrofused MDA-MB-231 breast cancer cell line with day-3 DCs derived from peripheral blood monocytes, and explored the biological characteristics of fusion vaccine and its anti-tumor effects in vitro. Day-3 mature DCs were generated from day-2 immature DCs by adding cocktails composed of TNF-α, IL-1β, IL-6 and PEG_2_. Day-3 mature DCs were identified and electofused with breast cancer cells to generate fusion vaccine. Phenotype of fusion cells were identified by fluorescence microscope and flow cytometer. The fusion vaccine was evaluated for T cell proliferation, secretion of IL-12 and IFN-γ, and induction of tumor-specific CTL response. Despite differences in morphology, day-3 and day-7 DC expressed similar surface markers. The secretion of IL-12 and IFN-γ in fusion vaccine group was much higher than that in the control group. Compared with control group, DC-tumor fusion vaccine could better stimulate the proliferation of allogeneic T lymphocytes and kill more breast cancer cells (MDA-MB-231) in vitro. Day-3 DCs had the same function as the day-7 DCs, but with a shorter culture period. Our findings suggested that day-3 DCs fused with whole apoptotic breast cancer cells could elicit effective specific antitumor T cell responses in vitro and may be developed into a prospective candidate for adoptivet immunotherapy.

## Introduction

Breast cancer has always been recognized as a major culprit of female mortality [1], with an incidence of nearly 80 to 100 out of every 100, 00 women in UK and USA. Similar data were also reported from Asian countries. Luckily, major advances in breast cancer treatment have been achieved over the last 20 years, leading to significant improvement in the rate of disease-free survival. Among various clinical approaches, a cancer vaccine would have important advantages over other available therapies for breast cancer. It could be easily administered and would be predicted to have no significant side effects because it would be extremely specific [2].

The basic concept of developing a vaccine for specific tumor cell antigens is uncomplicated, but the development of effective cancer vaccines for solid tumors has met with limited success. This is exactly the case in breast cancer. Although there are many potential explanations for this incomplete success, the major underlying challenge is that breast cancer cells have many subgroups that vary in morphology, biology, behavior and response to therapy. One subtype of the breast cancer is triple-negative breast cancer (TNBC), with the characteristics of estrogen receptor (ER) negative, progesterone receptor (PR) negative and human epidermal growth factor receptor-2(Her-2). TNBC's aggressive clinical behavior results in its unfavorable reaction to endocrine therapy and anti-Her2 targeted therapy, thus creating a niche for a more effective clinical solution.

Dendritic cells (DCs) are specialist antigen-presenting cells (APC) playing a pivotal role in immune sentinels as initiators of T-cell responses against tumors and microbial pathogens [3]. Upon stimulation with tumor associated antigen or bacterial products, DCs undergo a maturation process that causes upregulation of co-stimulatory molecules, high-level expression of major histocompatibility complex (MHC) and migration into secondary lymphoid organs where they prime naïve T cells [4], [5]. Because of the unique capacity to stimulate resting T cells, DCs are the most promising option for immunization protocols.

Among the various cellular sources, PBMC was more adopted than other sources such as cord blood and bone marrow to generate DCs because the monocytes can be easily obtained from peripheral blood in large numbers. [6], [7]

Currently, numerous protocols were developed to prepare mDCs varying in the time periods and the signals used for maturation in vitro. The traditional methods required about seven days of cell culture using the following protocol: 5 days to generate immature DCs with GM-CSF and IL-4, then 2 or 3 days to induce the maturation of DCs with microbial, proinflammatory, or T cell-derived stimuli. To generate DCs-based vaccine for rapid clinical trial use, shorter DCs differentiation protocols have been investigated. Previous studies indicated that mDCs could also be generated within 2 days using a maturation cocktail, including TNF-α, IL-1β, IL-6 and PEG_2_ [8]. These so called “fast DCs”, though exhibiting high surface expression of CD80, CD86, HLA-DR and producing high level of IL-12, demonstrated some impairment in migratory capacity [9], [10], [11]. Therefore, Maja Burdek and his colleagues improved this Day-2 “fast DCs” protocol to prepare young mDCs by extending the time period to 48 h, followed by addition of the maturation cocktail for another 24 h, giving a total culture period of 72 h [12]. The Day-3 protocol was not only more time saving and cost effective for DC-based vaccine development, but also led to a higher yield of cells with greater viability and the equal capacity to activate CTLs.

DCs can acquire target antigens through tumor antigen peptide by breaking tumor cells, co-culturing with tumor cells, or transfecting DCs by tumor antigen associated genes. However, the broken tumor cells may not provide sufficient antigens to DCs. It was even worse in tumor cells like TNBC, which lack ER, PR, and HER-2 typical to other breast cancer cells. Therefore, we assume that the protocol to fuse whole TNBC tumor cells with DCs could integrate the antigenicity of tumor cells and DCs functions of antigen presenting and T cells activating, thus achieving a stronger tumor killing effect.

In the present study, we performed a comparative investigation of day-3 and day-7 mDCs in terms of morphology, phenotype, and subsequently evaluated whether day-3 mDCs fused with apoptotic TNBC cells (MDA-MB-231) could elicit T cell responses of proliferation, cytotoxicity, and cytokine release against allogeneic tumor cells. Our aim was to obtain preclinical evidence for the potential efficacy of day-3 DC/tumor vaccine therapy that such an approach may hold key to the potential treatment with adoptive immunotherapy for patients with TNBC.

## Materials and Methods

We confirm that the use of human subjects was specifically approved by the Clinical Research Ethics Committee of the Third Affiliated Hospital, Sun Yat-sen University. Guangzhou Blood Center supplied the blood and recorded the informed consent. Before donating blood, the volunteers had known and agreed the content that the blood was going to use for clinical treatment and scientific research. We are sure that all the healthy volunteers agreed to participate in the study in verbal informed consent.

### Cell culture

Human TNBC cell line MDA-MB-231was purchased from the Sun Yat-Sen University Cancer Center (They obtained from American Type Culture Collection-ATCC). The cells were cultured in RPMI-1640 (purchased from Gibco CO., USA) containing 10% standard fetal bovine serum (Gibco CO., USA), 2 mM L-glutamine,100 U/ml penicillin, 100 µg/ml streptomycin, and incubated in an environment containing 5% CO_2_ at 37°C. The cell line was routinely tested for mycoplasma and found negative. Cells in logarithmic growth phase were chosen for experiment.

### Generation and culture of Day-3 and Day-7 DCs

Human peripheral venous blood was obtained from Guangzhou Blood Center and heparinized under aseptic condition. PBMC were isolated from peripheral blood by Ficoll-Hypaque gradient centrifugation and the lymphocytes separating solution purchased from Tianjin Hanyang Biologicals Technology Co., Ltd. was used. PBMCs were subsequently cultured in serum-free RPMI 1640 medium in 6-well plates at 37°C and 5%CO2 for 2 h. Non-adherent cells were removed by gently washing with phosphate-buffered saline (PBS) solution and used for T cell enrichment by nylon wool. Adherent cells were replenished with RPMI 1640 medium containing 100 ng/ml recombinant human granulocyte macrophage-colony stimulating factor (rhGM-CSF; PeproTech CO., USA) and 20 ng/ml recombinant human interleukin-4 (rhIL-4; PeproTech) for 2 days and subsequently incubated with a combination of proinflammatory mediators for 24 h (1000 U/ml TNF-α, 10 ng/ml IL-1β, 10 ng/ml IL-6, and 1 µM PGE_2_, PeproTech). To generate the Day-7 DCs, adherent cells were cultured for 5 days with GM-CSF and IL-4, followed by incubation with the same proinflammatory mediators for another 48 h.

### Electrofusion of DCs and tumor cells

Day-3 mature DCs were mixed with MDA-MB-231 at a ratio of 1∶1 in fusion medium, respectively. The tumor cells were treated with mitomycin C,50 µg/ml (NanJing KeyGEN Biotech Co., Ltd) at 37°Cfor 30 min. Fusion medium consisted of 280 mM glucose solution containing 0.1 mM Ca(CH_3_COO)_2_, 0.5 mM Mg(CH_3_COO)_2_, and 0.3% bovine serum albumin at 2∶1 ratio (PH = 7.3). After washed twice with the fusion medium, the mix cells were resuspended in 1 ml fusion medium at a concentration of 1×10
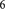
cells in an electroplated cuvette (Bio-Rad, CO., USA). Electrofusion was carried out using a fusion chamber connected to a pulse generator (Gene Pulser Xcell-TY8488, Bio-Rad). Cell alignment was first induced by dielectrophoresis with an alternating-current pulse of 200 V/cm for 10 s. Subsequently, cell fusion was triggered by application of direct current pulse of 1000 V/cm for 20 µs. The cuvette was left to stand for at least 5 min before cells were removed in a series of PBS rinses. The fusion mixture was resuspended in RPMI-1640 with 10% FBS and incubated overnight. After rinsing, adherent cells containing fusion hybrids were harvested. Fusion cells were purified by Flow Cell Sorter.

### Identification of fusion cells

Fusion cells were identified by the morphology, fluorenscence microscope and flow cytometry. The morphology was carried out by inverted microscope; tumor cells were pre-labeled with fluorescein isothiocyanate (FITC)-conjugated anti-Muc1 (BD CO., USA). The hybrid cells were incubated with phycoerythrin (PE)-conjugated anti-CD11c (BD) for 30 min at 4°C. Cells were washed, fixed and analyzed by fluorenscence microscope and FACScan (BD) with CellQuest analysis software.

### Flow cytometry analysis

Cells were washed and incubated with FITC-conjugated antibody against CD83,CD86, or Muc-1 and PE-conjugated antibody against CD11c and HLA-DR (BD) for 30 min at 4°C. After washing with cold PBS, cells were fixed with 2% paraformaldehyde and analyzed by FACScan.

### Mixed lymphocyte reactions induced by Day-3 and Day-7 DC fusion vaccine

Nonadherent PBMCs, as mentioned above, were purified through nylon wool to remove antigen-presenting cells and B cells. Then T lymphocytes were cultured in RPMI-1640 containing rhIL-2 20 ng/ml, to prepare as the reactive cells. Day-3 or Day-7 DC/MDA-MB-231 fusion cells, Day-3 DC mixed with MDA-MB-231, and Day-3 DC alone were cultured at the same time. These four groups of cells were added into 96-well plates with T cells at a ratio of 5∶1, 10∶1, and 20∶1, three wells for each group. T cell control group and culture medium blank control group were established. After 72 h of culture, 10 µl Cell Counting Kit (CCK8; NanJing KeyGEN) solution was added successively. Absorbance OD value was detected by enzyme- labeled instrument at 570 nm and the stimulation index (SI) obtained for triplicate assays.

### Cytokine detection by ELISA

T lymphocytes enriched by nylon wood were cultured in 24 well plates with complete medium containing rhIL-2 20 ng/ml, prepared as effector cells. DC/MDA-MB-231 fusion cells, DC mixed with MDA-MB-231, and DC alone were plated with the effector T cells at a ratio of 10∶1 (1×10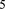 stimulating cells/1×10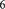 effector cells) and cultured for 7 days at 37°C and 5% CO2. The effector cells were serially stimulated a total of three times. Five days after the third stimulation, supernatant levels of IFN-γ and IL-12 were measured with human IFN-γ and IL-12 ELISA detection kits (NanJing KeyGEN) according to the manufacturer's instructions. Cytokine release was reported as mean picograms ± SEM of IFN-γ and IL-12 for triplicate assays.

### Cytotoxicity assay

For the induction of tumor-specific CTL, T lymphocyte were incubated with stimulators (Day-3 or Day-7 DC/MDA-MB-231 fusion cells, Day-3 DCs mixed with tumor cells, Day-3 DCs alone) at a ratio of 10∶1 in 24-well culture plates in RPMI 1640 medium containing IL-2 for 5 days at 37°C, 5% CO_2_. The CTLs were harvested and incubated with target cells (MDA-MB-231, MCF-7, PC-3) at a ratio of 5∶1, 10∶1, 20∶1 in 96-microwell plates at 37°C, 5% CO_2_ for 72 h as the reactive cell group, respectively. Concurrently, pure CTL group, pure target cell control group and pure culture solution blank control group were established. In the last 4 h, 10 µl CCK8 solution was added. Absorbance OD value was detected by enzyme-labeled instrument at 570 nm. The following formula was used to calculate killing rate, killing rate  =  [1-(OD value of reactive cell well-OD value of effector cell well)/(OD value of target cell well)]×100%.

### Statistical analysis

The statistical differences between experimental groups and controls were determined by the Student's t-test and were considered significant if two-tailed P values were less than 0.05.

## Results

### Morphology and phenotype of Day-3 and Day-7 DCs

**Figure 1:**
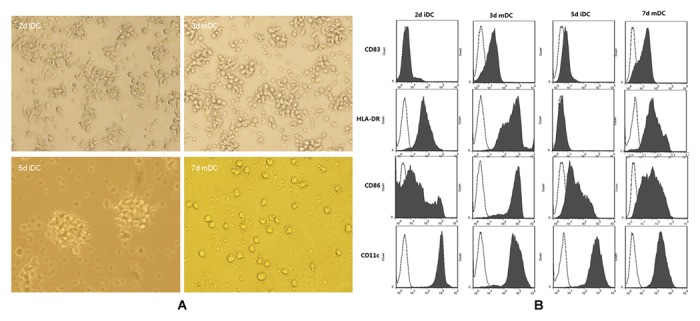
Morphology and phenotype of immature and mature DCs. PBMC were isolated from peripheral blood by Ficoll-Hypaque gradient centrifugation and subsequently cultured in serum-free RPMI 1640 medium in 6-well plates at 37°C and 5%CO2 for 2 h. Non-adherent cells were removed by gently washing with PBS. Adherent cells were replenished with RPMI 1640 medium containing GM-CSF 100 ng/ml and IL-4 20 ng/m. (**A**) Morphology of Day-2 imDC, Day-3 mDC, Day-5 imDC and Day-7 mDC. (**B**) Phenotype of Day-2 imDC, Day-3 mDC, Day-5 imDC and Day-7 mDC Day-3 and Day-7 DC.

All types of DCs were generated using monocytes obtained via plate adherence of freshly isolated PBMC of healthy donors. In all experiments, the four proinflammatory mediators were used for DC maturation. The Day-3 DCs were generated within 72 h, whereas Day-7 DCs were generated within one week. The different DC types were analyzed via flow light microscopy and cytometry (Figure 1A and 1B). It was shown that Day-3 mDC were similar in size to Day-2 iDC, while Day-7 mDC were larger than Day-6 iDC. It was noticeable that Day-7 mDC were much larger and indicated longer, better cytoplasmatic protrusions than Day-3 mDC. Moreover, Day-3 mDC displayed a higher yield and viability compared to Day-7 mDC (data not shown). As for the phenotype, DCs were stained with monoclonal antibodies specific for cell surface molecules typically expressed on iDC and mDC and subsequently analyzed via flow cytometry. Day-2 iDC and Day-6 iDC displayed no CD83 and low expression of CD86. Typically, Day-3 and Day-7 mDC showed high expression of CD83, constimulatory molecules CD86, as well as other molecules that are important for the function of mDCs, including CD11c and HLA-DR. Despite the shorter culture time, 3 d mDCs often expressed higher levels of activation markers, which was comparable to that of Day-7 mDCs.

### Characteristics of Day-3 DC/TNBC fusion cells

**Figure 2:**
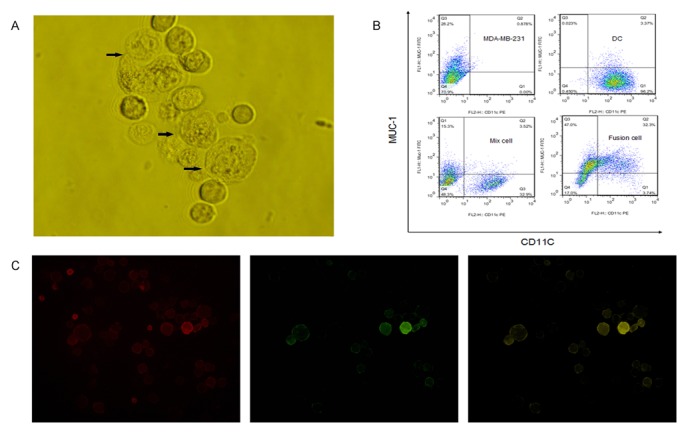
Identification of fusion vaccine. Day-3 mature DCs were electrofused with MDA-MB-231 to generate fusion vaccine. The fusion vaccine was identified from different way. (**A**) Morphology of fusion vaccine. (**B**), (**C**) Tumor cells were pre-labeled with fluorescein isothiocyanate (FITC)-conjugated anti-Muc1. The hybrid cells were incubated with phycoerythrin (PE)-conjugated anti-CD11c. Cells were washed, fixed and analyzed by fluorenscence microscope and FACScan with CellQuest analysis software.

Day-3 DCs were generated from PBMC and cultured in GM-CSF/IL-4 medium with the following stimulation of four proinflammatory mediators. Then Day-3 DCs were fused with apoptotic TNBC cell line MDA-MB-231 via electrofusion. The fusion cells were observed under light microscope. We can see that DCs and tumor cells were fused, with cytomembrane and cytoplast partially fused. The karyons of parent cells were close to each other, but without the sign of fusion (Figure 2A). The fluorenscence microscopic results demonstrated that tumor cells stained with FITC-conjugated Muc1 appearedgreen fluorenscent and DCs stained with PE-conjugated CD11c were red fluorenscent. The fusion cells consisting of DCs and tumor cells presented yellow fluorenscent in areas of colocalization (Figure 2B). FACS analyses to assess fusion cells demonstrated that DCs expressed CD11c molecules and were negative for Muc-1 tumor antigen, whereas MDA-MB-231 expressed Muc-1 tumor antigens and were negative for CD11c molecule. DC/MDA-MB-231 fusion cells both expressed CD11c and Muc-1. The fusion efficiency was about 30% after subtracting the double positive cells from DC mixed with tumor cells (Figure 2C). Fusion cells were purified by Flow Cell Sorter for the coexpression of CD11c and Muc-1. These results provided unequivocal evidence that the fusion cells possessed the phenotypic properties of their parent cells.

### T-cell proliferation assay

**Figure 3:**
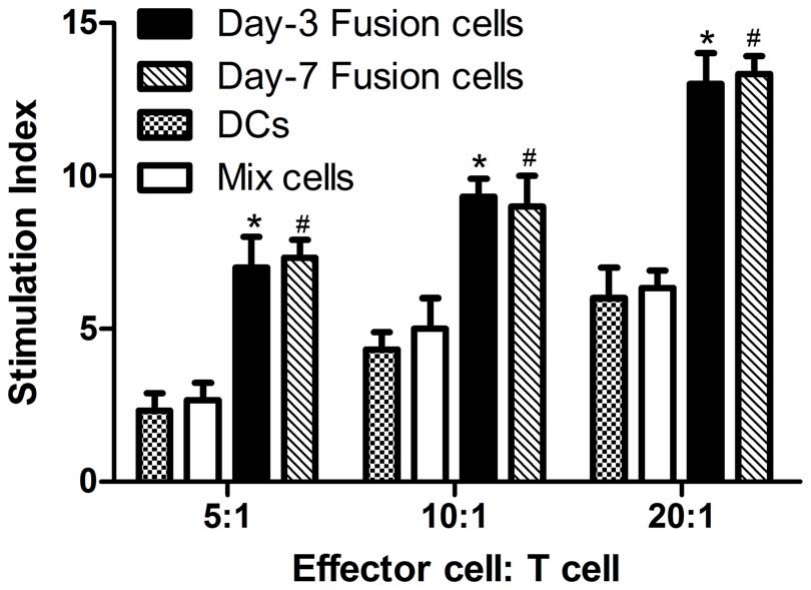
The proliferation of T cells stimulated by fusion cells. T lymphocytes were cultured in RPMI-1640 containing rhIL-2 20 ng/ml, to prepare as the reactive cells. Day-3 and Day-7 fusion cells, mixed cells, and DC alone were cultured at the same time. These four groups of cells were added into 96-well plates with T cells at a ratio of 5∶1, 10∶1, and 20∶1, three wells for each group. T cell control group and culture medium blank control group were established. Each bar represents the mean ± standard error of the mean.* Significantly different from the DC and mix cell groups, *P*<0.05, #No difference between Day-3 and Day-7 fusion vaccine.

T cells were cocultured with fusion cells to determine whether Day-3 DC/MDA-MB-231 fusion cells are effective in stimulating T cell proliferation as Day-7 DC fusion cells. In the control groups, Day-7 DC/MDA-MB-231 fusion cells, Day-3 DCs mixed with and without MDA-MB-231 were also cocultured with T cells simultaneously. Similar significant T-cell proliferation was induced by Day-3 and Day-7 DC/MDA-MB-231 fusion cells (*P*>0.05), and to a much lesser extent, by Day-3 DCs mixed with MDA-MB-231 and Day-3 DCs alone (*P*<0.05). No statistically significant differences were obtained between Day-3 DCs mixed with MDA-MB-231 group and Day-3 DCs alone group (*P*>0.05). DC/MDA-MB-231 fusion cells at a ratio of 20∶1 induced more significant T cells proliferation than that at ratios of 5∶1 and 10∶1 (*P*<0.05) (Figure 3).

### Secretion of IFN-γ, IL-12 in DC/MDA-MB-231fusion cells cocultured with T cells

**Figure 4:**
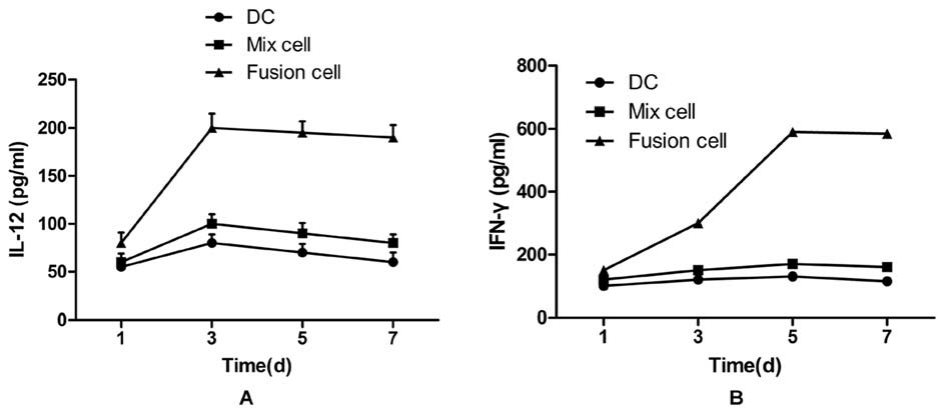
The secretion of IL-12 and IFN-γ in the supernatant of each group. Fusion cells, mixed cells, and DCs alone were plated with the effector T cells at a ratio of 10∶1. The effector cells were serially stimulated a total of three times. Five days after the third stimulation, supernatant levels of IFN-γ and IL-12 were measured with human IFN-γ and IL-12 ELISA detection kits. Cytokine release was reported as mean picograms ± SEM of IFN-γ and IL-12 for triplicate wells. * Significantly different from the control groups, *P*<0.05.

Cytokine secretion was analyzed to determine T cell activation and to explore the relationship between IL-12 and IFN-γ. Supernatant of fusion cells group was collected at indicated time points to detect the expression of IL-12 and IFN-γ. The secretion peak of IL-12 in DCs (81.25±8.54 pg/ml) and Mix cells groups (103.18±9.12 pg/ml) was on the third day, then the level was slowly declined (Figure 4A). In contrast, the peak level secretion of IL-12 detected in fusion group (204.87±10.03 pg/ml) was on the third day, but it remained stable without obvious decline. The secretion level of IL-12 in fusion cell group was higher than that in DCs and Mix cells group (*P*<0.05). In the fusion group, the secretion peak of IFN-γ was on the fifth day and the level in fusion group (591.14±11.68 pg/ml) was higher than the contrast groups (DC132.76±9.12 pg/ml, Mix cells168.43±10.75 pg/ml) (Figure 4B).

### Induction of antigen-specific CTLs by fusion vaccine

**Figure 5:**
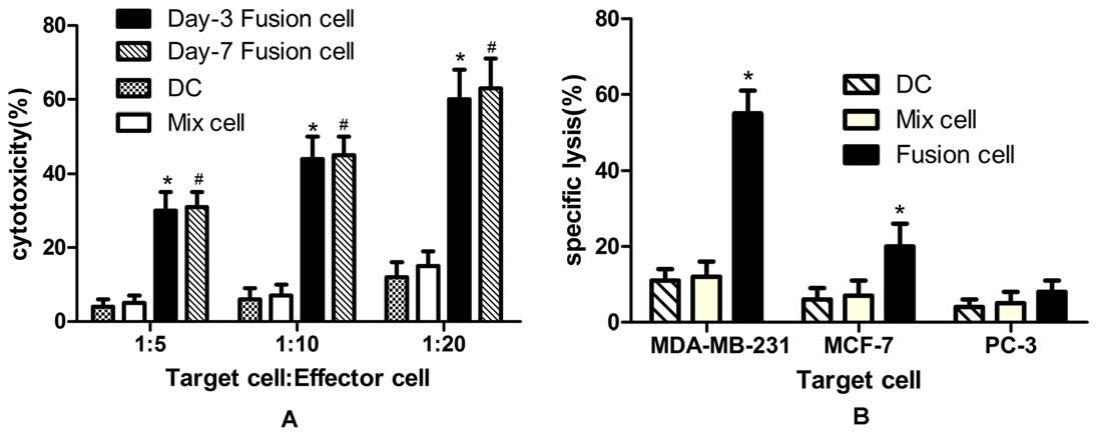
Antigen-specific CTLs by fusion vaccine. (**A**) The CTLs induced by Day-3 or Day-7 fusion vaccine, mix cell and DC alone were harvested and incubated with target cells (MDA-MB-231) at a ratio of 5∶1, 10∶1, 20∶1 in 96-microwell plates. (B) The CTLs induced by Day-3 fusion vaccine, mix cell and DC alone were harvested and incubated with target cells (MDA-MB-231, MCF-7, PC-3). The following formula was used to calculate killing rate, killing rate  =  [1-(OD value of reactive cell well-OD value of effector cell well)/(OD value of target cell well)]×100%. * Significantly different from the DC and mix cell groups, *P*<0.05, #No difference between Day-3 and Day-7 fusion vaccine.

We first tested cytotoxic activity against MDA-MB-231, using CTLs induced by fusion vaccine. The results showed that the Day-3 fusion vaccines were able to elicit a more impressive CTL response against MDA-MB-231 and the cytotoxic activity was as strong as Day-7 fusion vaccine. The cytotoxic activity was more intense when the effector- target ratio increased. In contrast, DCs and Mix cells group did not induce a strong CTL response (Figure 5A). Then we determined whether DCs electrofused with MDA-MB-231 can elicit specific CTL with cytotoxic activity against MDA-MB-231 in vitro. The results displayed that CTLs stimulated by DC/MDA-MB-231fusion vaccine were effective in inducing lysis of MDA-MB-231 cells. In contrast, cytotoxic activity against MCF-7 cells and PC-3 cells failed to generate by MDA-MB-231 specific CTLs (Figure 5B). In brief, these results indicated that the CTLs stimulated by DC/MDA-MB-231 fusion cells had specific antitumor efficacy against MDA-MB-231 cells.

## Discussion

In the present study, we fused DCs with TNBC tumor cells to generate whole antigen vaccine. Previous investigations reported that DCs could present antigens by means of MHC molecules and co-stimulating signals, thus being recognized as the most powerful antigen presenting cells (APC), initiating body anti-tumor immunity reactions through naive T cells activation and proliferation [13]. These unique functions of DCs could be applied into anti-tumor immunotherapy [14], [15] and had met with some success in malignant melanoma[16], renal cell carcinoma[17], breast cancer[18] etc.

DCs maturity was also reported to relate to their functions. Immature DCs (iDC) are located in peripheral tissues and take up antigens via phagocytosis, macropinocytosis or receptor-mediated endocytosis. Antigens taken by iDCs would be processed and presented as antigen-derived peptides on their MHC molecules. The presentation of antigens by iDCs can lead to deletion of T cells, T cell anergy or induction of TGF-β, and IL-10 secreted by T regulatory cells [19]. With antigens uptake, iDCs could be converted to a mature phenotype, characterized by the upregulation of immunophenotyping, such as CD83, CD86, CD11c, HLA-DR[20].

In the present study, Day-3 DC preparation protocols were developed to speed up DC vaccine preparationand to reduce labor requirements and costs[12]. During the preparation,we added maturation cocktail to the iDC cultures on the second day, and the mature DCs (mDC) were yielded on Day-3.We observed that Day-3 mDCs were more robust cells, reflected in a lower granularity and smaller morphology, resulting in a higher yield of cells with greater viability. The difference in morphology raised the issue whether Day-3 mDCs would be different from Day-7 mDCs in immunophenotyping. The results showed that both types of mDCs expressed similar levels of important molecules of CD11c, CD83, CD86 and HLA-DR.

Previous investigations reported such approaches to load DCs with target antigens as acquiring tumor antigen peptide by breaking tumor cells, stimulating DCs through co-culturing with tumor cells, transfecting DCs by tumor antigen associated genes [21], [22], [23]. However, DC-based immumotherapy has met with only limited success due to the following factors: the tolerant environment of the tumor, the limited availability of known tumor-associated antigens, and the insufficient numbers and the functional defect of DCs from patients [24]. The fragmented antigens might be particularly responsible for this limitation. It was even worse in tumor cells like TNBC, which have no special antigen or antigen gene and without expression of ER, PR, and HER2. Fortunately, with the development of cell fusion technique in more recent years, researchers have attempted to fuse DCs with tumor cells to generate the fusion vaccine, integrating the antigenicity of tumor cells and DCs functions of antigen presenting and T cells activating [25].

In the present study, we fused DCs with triple-negative breast cancer (TNBC) cells as the whole antigen to generate the vaccine to target TNBC cells through T cells mediation. Day-3 mDCs were fused with TNBC cells (MDA-MB-231) via electrofusion to make TNBC specific vaccine. We found that fusion cells possessed the phenotype of their parent cells. The hybrids were capable of inducing a lymphocyte proliferation response, which was important in anti-tumor immunity.

IL-12 was shown to play a pivotal role in promoting the polarization of Th1 cells and promoting the secretion of IFN-γ, IL-2 and TNF by CD8+CTL, which were essential in the induction and maintenance of CTL. IFN-γ secreted by Th1 or CD8+CTL cells has a powerful effect in enhancing the ability of DCs to produce IL-12, and IL-12 also enhances the production of IFN-γ, thus creating a positive reinforcement loop [26]. In the presenting study, the secretion of IL-12 in culture medium supernatants increased gradually and reached the highest level on day-3 after fusion, and then the secretion level tended to be stable, which was significantly different from the secretion in the control group in which the secretion of IL-12 gradually decreased after reaching the highest level on day-3. Meanwhile, the detection of secretion of IFN-γ in the upper solution found that the highest level of IFN-γ was on day-5 in the fusion group and the secretion level tended to be stable while the secretion in the control group decreased on day-3. The highest secretion level of IFN-γ was later than that of IL-12, indicating that the IL-12 may facilitate the secretion of IFN-γ. Altogether, these results demonstrated that the fusion cells could up-regulate and guarantee continuous IL-12 secretion and then promote the secretion of IFN-γ to maintain the antitumor activity.

Our next approach was to determine the specific cytotoxic activity of Day-3 fusion vaccine. The results revealed that Day-3 DCs fused with apoptotic tumor cells could elicit the similar cytotoxicity with Day-7 DC/tumor vaccine and more intense antitumor activity than the control groups. To confirm the antigen-specific of CTL, we used the TNBC antigen-sensitized CTLs to co-culture with MDA-MB-231, MCF-7 and PC-3, and discovered the higher CTL activity against MDA-MB-231 than against MCF-7 and PC-3. Moderate killing to MCF-7 might reveal their possession of some common unidentified antigens with MDA-MB-231.

### Conclusion

Our study indicated that it is feasible to prepare TNBC specific vaccine by electrofusion with peripheral blood Day-3 DCs. This strategy of generating Day-3 DCs not only reduces cost, labor, and time for DC development, but also represents a novel model to generate cancer vaccines.

## References

[1] Siegel R, Naishadham D, Jemal A (2013) Cancer statistics. CA Cancer J Clin 62: 10–29. 10.3322/caac.20138 22237781

[2] Mittendorf EA, Peoples GE, Singletary SE (2007) Breast cancer vaccines promise for the future or pipe dream? Cancer 110: 1677–1686. 17763371 10.1002/cncr.22978

[3] Kapsenberg ML (2003) Dendritic-cell control of pathogen-driven T-cell polarization. Nat Tev Immunol 3: 984–993. 10.1038/nri1246 14647480

[4] Adams S, O'Neill DW, Bhardwaj N (2005) Recent advances in dendritic cell biology. J. Clin. Immunol 25: 87–98. 10.1007/s10875-005-2814-2 15821885

[5] Cella M, Sallusto F, Lanzavecchia A (1997) Origin, maturation and antigen presenting function of dendritic cells. Curr. Opin. Immunol 9: 10–16. 10.1016/s0952-7915(97)80153-7 9039784

[6] Tuyaerts S, Aerts JL, Corthals J, Neyns B, Heirman C, et al (2007) Current approaches in dendritic cell generation and future implications for cancer immunotherapy. Cancer Immunol. Immunother 56: 1513–1537. 10.1007/s00262-007-0334-z PMC11030932 17503040

[7] Campbell-Anson RE, Kentor D, Wang YJ, Bushnell KM, Li Y, et al (2008) A new approach for the large-scale generation of mature dendritic cells from adherent PBMC using roller bottle technology. J Immune Based Ther Vaccines 6: 1. 18321390 10.1186/1476-8518-6-1 PMC2292722

[8] Dauer M, Obermaier B, Herten J, Haerle C, Pohl K, et al (2003) Mature dendritic cells derived from human monocytes within 48 hours: a novel strategy for dendritic cell differentiation from blood precursors. J Immunol 170: 4069–4076. 12682236 10.4049/jimmunol.170.8.4069

[9] Dauer M, Schad K, Herten J, Junkmann J, Bauer C, et al (2005) FastDC derived from human monocytes within 48 h effectively prime tumor antigen-specific cytotoxic T cells. J Immunol Methods 302: 145–155. 15992809 10.1016/j.jim.2005.05.010

[10] Jarnjak-Jankovic S, Hammerstad H, Saeboe-Larssen S, Kvalheim G, Gaudernack G (2007) A full scale comparative study of methods for generation of functional dendritic cells for use as cancer vaccines. BMC Cancer 7: 119. 17608923 10.1186/1471-2407-7-119 PMC1931601

[11] Randolph GJ, Beaulieu S, Lebecque S, Steinman RM, Muller WA (1998) Differentiation of monocytes into dendritic cells in a model of transendothelial trafficking. Science 282: 480–483. 9774276 10.1126/science.282.5388.480

[12] Burdek M, Spranger S, Wilde S, Frankenberger B, Schendel DJ, et al (2010) Three-day dendritic cells for vaccine development: Antigen uptake, processing and presentation. J Transl Med 8: 90. 20920165 10.1186/1479-5876-8-90 PMC2955579

[13] Curigliano G, Spitaleri G, Dettori M, Locatelli M, Scarano E, et al (2007) Vaccine immunotherapy in breast cancer treatment: promising, but still early. Expert Rev Anticancer Ther 7: 1225–1241. 17892423 10.1586/14737140.7.9.1225

[14] Zhang Y, Ma B, Zhou Y, Zhang M, Qiu X, et al (2007) Dendritic cells fused with allogeneic breast cancer cell line induce tumor antigen-specific CTL responses against autologous breast cancer cells. Breast Cancer Res Treat 105: 277–286. 17187233 10.1007/s10549-006-9457-8

[15] Delirezh N, Moazzeni SM, Shokri F, Shokrgozar MA, Atri M, et al (2009) Autologous dendritic cells loaded with apoptotic tumor cells induce T cell-mediated immune responses against breast cancer in vitro. Cell Immuno 257: 23–31. 10.1016/j.cellimm.2009.02.002 19306994

[16] Trefzer U, Herberth G, Wohlan K, Milling A, Thiemann M, et al (2005) Tumour-dendritic hybrid eell vaccination for the treatment of patients with malignant melanoma: immunological effects and clinical results. Vaccine 23: 2367–2373. 15755630 10.1016/j.vaccine.2005.01.081

[17] Avigan DE, Vasir B, George DJ, Oh WK, Atkins MB, et al (2007) Phase I/II study of vaccination with electrofused allogeneic dendritic cells/autologous tumor-derived cells in patients with stage IV renal cell carcinoma. J lmmunother 30: 749–761. 10.1097/CJI.0b013e3180de4ce8 17893567

[18] Avigan D, Vasir B, Gong J, Borges V, Wu Z, et a1 (2004) Fusion cell vaccination of patients with metastatic breast and renal cancer induces immunological and clinical responses. Clin Cancer Res 10: 4699–4708. 15269142 10.1158/1078-0432.CCR-04-0347

[19] Jonuleit H, Schmitt E, Schuler G, Knop J, Enk AH (2000) Induction of interleukin10-producing, nonproliferating CD4(+) T cells with regulatory properties by repetitive stimulation with allogeneic immature human dendritic cells. J Exp Med 192: 1213–1222. 11067871 10.1084/jem.192.9.1213 PMC2193357

[20] Fong L, Engleman EG (2000) Dendritic cells in cancer immunotherapy. Annu Rev Immunol 18: 245–273. 10837059 10.1146/annurev.immunol.18.1.245

[21] Bohnenkamp HR, Coleman J, Burchell JM, Taylor-Papadimitriou J, Noll T (2004) Breast carcinoma cell lysate-pulsed dendritic cells cross-prime MUC1-specific CD8+ T cells identified by peptide-MHC-class-I tetramers. Cell Immunol 231: 112–125. 15919376 10.1016/j.cellimm.2004.12.007

[22] Ma W, Smith T, Bogin V, Zhang Y, Ozkan C, et al (2011) Enhanced presentation of MHC class Ia, Ib and class II-restricted peptides encapsulated in biodegradable nanoparticles: a promising strategy for tumor immunotherapy. J Transl Med 9: 34. 21450109 10.1186/1479-5876-9-34 PMC3078865

[23] Bonehill A, Heirman C, Tuyaerts S, Michiels A, Breckpot K, et al (2004) Messenger RNA-electroporated dendritic cells presenting MAGE-A3 simultaneously in HLA class I and class II molecules. J Immunl 172: 6649–6657. 10.4049/jimmunol.172.11.6649 15153480

[24] Benencia F, Sprague L, McGinty J, Pate M, Muccioli M (2012) Dendritic cells the tumor microenvironment and the challenges for an effective antitumor vaccination. J Biomed Biotechnol 2012: 425476. 22505809 10.1155/2012/425476 PMC3312387

[25] Avigan DE, Vasir B, George DJ, Oh WK, Atkins MB, et al (2007) Phase I/II study of vaccination with electrofused allogeneic dendritic cells/autologous tumor-derived cells in patients with stage IV renal cell carcinoma. J Immunother 30: 749–776. 17893567 10.1097/CJI.0b013e3180de4ce8

[26] Trinchieri G (1998) Proinflammatory and immunoregulatory functions of interleukin-12. Int Rev Immunol 16: 365–369. 9505196 10.3109/08830189809043002

